# Evaluation of two novel tablet formulations of artemether-lumefantrine (Coartem®) for bioequivalence in a randomized, open-label, two-period study

**DOI:** 10.1186/1475-2875-12-312

**Published:** 2013-09-08

**Authors:** Gilbert Lefèvre, Prafulla Bhad, Jay Prakash Jain, Sampath Kalluri, Yi Cheng, Hardik Dave, Daniel S Stein

**Affiliations:** 1 Novartis Institutes for BioMedical Research, Postfach, CH-4002, Basel, Switzerland; 2 Novartis Institutes for BioMedical Research, Inc, Cambridge, USA; 3 Novartis Healthcare Private Limited, Hyderabad, India; 4 Beijing Novartis Pharma Co. Ltd, Shanghai, China; 5 Veeda Clinical Research Pvt. Ltd, Ahmedabad, India; 6 Novartis Institutes for BioMedical Research, East Hanover, NJ, USA

**Keywords:** Artemether, Lumefantrine, Coartem, Novel fixed-dose formulation, Bioequivalence

## Abstract

**Background:**

Artemether-lumefantrine (Coartem®; AL) is a standard of care for malaria treatment as an oral six-dose regimen, given twice daily over three days with one to four tablets (20/120 mg) per dose, depending on patient body weight. In order to reduce the pill burden at each dose and potentially enhance compliance, two novel fixed-dose tablet formulations (80/480 mg and 60/360 mg) have been developed and tested in this study for bioequivalence with their respective number of standard tablets.

**Methods:**

A randomized, open-label, two-period, single-dose, within formulation crossover bioequivalence study comparing artemether and lumefantrine exposure between the novel 80/480 mg tablet and four standard tablets, and the novel 60/360 mg tablet and three standard tablets, was conducted in 120 healthy subjects under fed conditions. Artemether, dihydroartemisinin, and lumefantrine were measured in plasma by HPLC/UPLC-MS/MS. Pharmacokinetic (PK) parameters were determined by non-compartmental analyses.

**Results:**

Adjusted geometric mean AUC_last_ for artemether were 345 and 364 ng·h/mL (geometric mean ratio (GMR) 0.95; 90% CI 0.89-1.01) and for lumefantrine were 219 and 218 μg·h/mL (GMR 1.00; 90% CI 0.93-1.08) for 80/480 mg tablet *versus* four standard tablets, respectively. Corresponding C_max_ for artemether were 96.8 and 99.7 ng/mL (GMR 0.97; 90% CI 0.89-1.06) and for lumefantrine were 8.42 and 8.71 μg/mL (GMR 0.97; 90% CI 0.89-1.05). For the 60/360 mg tablet *versus* three standard tablets, adjusted geometric mean AUC_last_ for artemether were 235 and 231 ng·h/mL (GMR 1.02; 90% CI 0.94-1.10), and for lumefantrine were 160 and 180 μg·h/mL (GMR 0.89; 90% CI 0.83-0.96), respectively. Corresponding C_max_ for artemether were 75.5 and 71.5 ng/mL (GMR 1.06; 90% CI 0.95-1.18), and for lumefantrine were 6.64 and 7.61 μg/mL (GMR 0.87; 90% CI 0.81-0.94), respectively. GMR for C_max_ and AUC_last_ for artemether and lumefantrine for all primary comparisons were within the bioequivalence acceptance criteria (0.80-1.25). In addition, secondary PK parameters also met bioequivalence criterion.

**Conclusion:**

Both of the novel artemether-lumefantrine tablet formulations evaluated are bioequivalent to their respective standard Coartem® tablet doses. These novel formulations are easy to administer and may improve adherence in the treatment of uncomplicated malaria caused by *Plasmodium falciparum*.

**Trial registration:**

Clinical trial registration number: CTRI/2011/12/002256

## Background

An estimated 216 million episodes of malaria were reported in 2010, of which approximately 81% were in the African region, and approximately 91% of these were due to *Plasmodium falciparum*[1]. Artemisinin-based combination therapy (ACT) is currently the best available treatments and recommended by the World Health Organization (WHO) [2] as the first-line treatment for uncomplicated malaria caused by *P*. *falciparum*; they are known to improve cure rates, reduce the chances of relapse (recrudescence and re-infection), reduce the development of resistance, and may decrease transmission of drug-resistant parasites [3-6]. Artemether-lumefantrine (AL; Coartem®) is the first fixed-dose ACT prequalified by WHO and it has been subsequently adopted by many countries as first-line treatment for uncomplicated *P*. *falciparum* malaria or mixed infections (*P*. *falciparum* and *Plasmodium vivax*). In clinical studies, AL has consistently demonstrated good efficacy (cure rate) and safety profiles [7].

AL is recommended to be given with food as a six-dose regimen that uses a fixed-dose combination tablet of 20 mg artemether and 120 mg lumefantrine. The regimen is given orally twice daily over three days with one to four standard tablets (20/120 mg) per dose, depending on patient’s body weight. For adults and children weighing ≥ 35 kg, each dose amounts to 80 mg artemether/480 mg lumefantrine (i.e., four tablets); for children weighing ≥ 25 kg, each dose amounts to 60 mg artemether/360 mg lumefantrine (i.e., three tablets) [8]. In order to potentially enhance compliance and to reduce the pill burden, two novel fixed-dose tablet formulations, 80/480 mg tablet (80 mg artemether/480 mg lumefantrine) and 60/360 mg (60 mg artemether/360 mg lumefantrine), have been developed.

The objective of this study was to demonstrate the bioequivalence with respect to exposure of artemether and lumefantrine between the novel 80/480 mg tablet and four standard tablets, and between the novel 60/360 mg tablet and three standard tablets, when administered to healthy volunteers under fed conditions.

## Methods

This was an open-label, randomized, single-dose, two-period, within formulation crossover study in healthy subjects under fed conditions as per label, as food enhances the absorption of lumefantrine. The study consisted of a 21-day screening period, two baseline periods (one before each treatment period), two treatment periods and a wash-out period of a minimum of five weeks followed by a study completion evaluation after the 264-hours blood draw of the last treatment period. Subjects who met the eligibility criteria at screening were admitted to the study site approximately 12 h prior to dosing in each period for baseline evaluations. The design consisted of two separate crossover comparisons, one comparing the novel 80/480 mg tablet *versus* four standard tablets (each 20 mg artemether/120 mg lumefantrine) taken once, and the second comparing the novel 60/360 mg tablet *versus* three standard tablets (each 20 mg artemether/120 mg lumefantrine) taken once. Study medications were administered under the supervision of study centre personnel with 240 mL of water in the morning between 07:30 and 09:00 following a high-fat, high-calorie breakfast (carbohydrate = 65.2 g (260.8 cal), protein = 37.5 g (150 cal), fat = 64.5 g (580.5 cal)). Each subject's mouth was checked to ensure that the medication was swallowed. Incidental use of paracetamol was allowed and was documented.

### Subjects

Healthy male and female subjects of non-childbearing potential, aged 18 to 55 years, weighing at least 50 kg and with a body mass index (BMI) within the range of 18–30 kg/sq m, and in good health as determined by past medical history, physical examination, vital signs, electrocardiogram (ECG), and laboratory tests at screening, were eligible for enrolment in the study. Subjects were excluded for the following reasons: using any prescription drugs at the time of enrolment; using any other investigational drugs at the time of enrolment or within 30 days or five elimination half-lives of enrolment (whichever is longer); history of hypersensitivity to artemether, lumefantrine, or drugs of similar chemical classes; history of clinically significant cardiac disease or ECG abnormalities or prolonged QT syndrome; smoking more than five cigarettes per day and unable to refrain from smoking during the study; donation or loss of more than 400 mL of blood within eight weeks prior to initial dosing; significant illness within two weeks prior to initial dosing; recent or recurring autonomic dysfunction or acute/chronic bronchospastic disease; any medical condition which might significantly alter the absorption, metabolism or excretion of the drugs (such as liver, bowel or pancreatic disease); laboratory values outside the normal laboratory range at screening; history of immunodeficiency disease; positive Hepatitis B surface antigen or Hepatitis C result; history of drug or alcohol abuse within 12 months prior to dosing; chest X-Ray indicating evidence of an active pulmonary process; or logistical or custodial reasons. This study was conducted at Veeda Clinical Research, Ahmedabad, India. The protocol was approved by the independent ethics committee associated with the study centre. Eligible subjects were enrolled after written informed consent. The study was conducted in accordance with the Declaration of Helsinki and local applicable laws and regulations (clinical trial registration number: CTRI/2011/12/002256). The study design was in accordance with the guidelines on the investigation of bioequivalence (CPMP/EWP/QEP/1401/98 Rev.1/Corr-2010; Food and Drug Administration (FDA) and European Medicines Agency (EMA) Guidance for Industry on bioequivalence studies). The first subject was enrolled on 3 January, 2012 and the last subject completed the study on 11 April, 2012.

### Assessments

The primary objective was to demonstrate bioequivalence with respect to exposure of artemether and lumefantrine with respect to AUC_last_ (area under the plasma concentration time curve up to the last quantifiable concentration) and C_max_ (observed maximum plasma concentration following drug administration) between the novel 80/480 mg tablet and four standard AL tablets, and between the novel 60/360 mg tablet and three standard AL tablets when administered to healthy volunteers under fed conditions. Other objectives included determination of the single-dose PK of artemether, dihydroartemisinin (DHA; active metabolite), and lumefantrine from the respective formulations under fed conditions and comparison of the DHA exposure between the respective formulations. Safety endpoints included adverse event rates, laboratory assessments, vital signs, and electrocardiographic data.

For the bio-analysis in plasma of artemether and DHA, blood samples of 3 mL were collected at predefined time points - pre-dose (i.e., 0 h), 0.5, 0.75, 1, 1.5, 2, 3, 4, 6, 8, 10, 12, 16, and 24 h post-dose. For bio-analysis of lumefantrine, blood samples of 2 mL were collected at predefined time points - pre-dose (i.e., 0 h), 1, 2, 4, 5, 6, 8, 10, 12, 24, 36, 48, 72, 120, 168, 216, and 264 h post-dose. The method used for the determination of artemether and DHA was a validated reversed-phase HPLC with MS/MS detection and for lumefantrine was a validated reversed-phase UPLC with MS/MS detection. The HPLC method for artemether and DHA was linear in the range of 3 ng/mL to 360 ng/mL using 10 μL of human plasma, and for lumefantrine was 50 ng/mL to 20 μg/mL using 5 μL of human plasma. For artemether at different concentrations, the intra- and interday accuracy (%) and precision (co-efficient of variation, % CV) were as follows: 3.00 ng/mL - intraday: 3.67, 1.98, interday: -0.67, 7.72; 9.0 ng/mL - intraday: 0.22, 2.58, interday: 3.44, 4.97; 36.0 ng/mL - intraday: 6.11, 1.82, interday: 4,17, 3.63; 144 ng/mL - intraday: 6.25, 1.57, interday: 3.47, 3.54; and 288 ng/mL - intraday: 0.00, 2.27 interday 2.78, 4.53. For DHA at different concentrations intraday and interday accuracy and precision were as follows: 3.00 ng/mL - intraday: 4.67, 2.54, interday: -1.33, 7.70; 9.0 ng/mL - intraday: 2.00, 4.36, interday: 2.44, 4.51; 36.0 ng/mL - intraday: 0.56, 2.43, interday: 1.67, 3.74; 144 ng/mL - intraday: 0.00, 2.47, interday: 0.69, 2.89; and 288 ng/mL - intraday: -4.86, 1.91, interday: 0.35, 6.16. For lumefantrine at different concentrations intra- and interday accuracy (%) and precision (% CV) were as follows: 50 ng/mL - intraday: 4.60, 2.03, interday: 4.40, 1.93; 150 ng/mL - intraday: 0.00, 3.39, interday: 1.33, 2.93; 3.00 μg/mL intraday: -0.67, 1.34, interday: 3.00, 4.08; 7.50 μg/mL - intraday: 3.73, 1.65; interday: 4.13, 1.45; and 15 μg/mL - intraday: 4.00, 0.63, interday: 4.00, 0.61. The PK parameters AUC_last_, C_max_, AUC_inf_ (AUC extrapolated to infinity), T_max_ (time required to achieve maximum plasma concentration), and terminal elimination half-life (T_1/2_) were calculated from plasma concentration-time data of artemether, DHA, and lumefantrine using non-compartmental analysis with WinNonlin Professional (V 6.2). Linear trapezoidal-linear interpolation was used for calculation of AUC_last_. AUC_inf_ was calculated based on last observed concentration and terminal elimination rate constant (minimum of 3 data points were included in the estimation of terminal elimination rate constant excluding C_max_ and terminal ‘0’ concentrations). For profiles where % AUC_inf_ extrapolated was > 20%, AUC_inf_ was excluded from the calculations. The actual recorded sampling times were used for PK parameter calculations.

### Statistical analysis

AUC_last_ and C_max_ of artemether and lumefantrine were the primary PK parameters to assess bioequivalence. It was determined from historical data [8] that a sample size of 30 subjects per treatment sequence would be adequate to give power between 91 and 99%, such that the 90% confidence intervals (CIs) for the ratio of treatment geometric means for AUC_last_ and C_max_ would lie within the interval (0.8, 1.25). This estimate was based on the assumption that the true ratio between treatments for both AUC_last_ and C_max_ was 1. The within-subject co-efficient of variations for PK parameters (AUC_last_ and C_max_) of the two analytes after administration of intact tablet was assumed ranging from 27.04 to 36.76% (Data on file, Novartis Pharma AG. Study CCOA566B2102 Unpublished).

PK analyses were performed on all subjects with evaluable drug plasma data and with no major protocol deviations impacting on the PK data. Safety analyses were performed on all subjects who received at least one dose of study drug.

Descriptive statistics of analyte concentrations by sampling time point as well as PK parameters by treatment included mean, standard deviation (SD), % CV, median, minimum and maximum. Concentrations below lower limit of quantification (LLOQ) were treated as zero in summary statistics.

An analysis of variance (ANOVA) was performed on the log-transformed (natural base) data with sequence, treatment, period, and subject nested within sequence as fixed effects. Only subjects with PK data for both periods were included in the bioequivalence assessment. Estimates of the geometric mean ratios (80/480 mg tablet *versus* four standard tablets; 60/360 mg tablet *versus* three standard tablets) and their 90% CIs were obtained. The estimates and CIs were back-transformed to the original scale, giving the ratios of geometric means for the two treatments being compared together with 90% CIs for the ratios. Bioequivalence was concluded if the 90% CIs were entirely contained within the acceptance range (0.80, 1.25). Other parameters such as AUC_inf_ of artemether and lumefantrine, as well as AUC_last_, C_max_ and AUC_inf_ of DHA, were analysed using the same model. Safety assessments included the recording of adverse events (AEs; by system organ class and preferred terms) and serious adverse events (SAEs), with their severity and relationship to the study drug, the collection of clinical laboratory data for hematology, blood chemistry, urine analysis, vital signs and ECG evaluations.

## Results

### Subject disposition, demographics and baseline characteristics

A total of 120 subjects were enrolled and randomized in this study. Sixty subjects were randomized into the crossover period comparing the novel 80/480 mg tablet to the four standard AL tablets, and additional 60 subjects were randomized into the crossover period comparing the novel 60/360 mg tablet to the three standard AL tablets. Five subjects (two subjects in 80/480 mg tablet *versus* four standard tablets, and three subjects in 60/360 mg AL tablet *versus* three standard tablets) did not complete the study. Two subjects were discontinued due to adverse events, one subject withdrew consent, one subject did not report for period 2, and one subject did not complete high-fat high-calorie breakfast before the dosing as mandated for evaluations.

All 120 subjects were included for PK analyses and safety analyses; 114 subjects were included in the bioequivalence assessment. The baseline demographics and clinical characteristics are presented in Table 1. Male subjects of Indian origin (age range 19–53 years) participated in the study.

**Table 1:** Subject demographics and baseline characteristics

		**80/480 mg and four standard tablets**			**60/360 mg and three standard tablets**		
		**Sequence**			**Sequence**		
		**1a**	**2a**	**Total**	**1b**	**2b**	**Total**
		**N = 30**	**N = 30**	**N = 60**	**N = 30**	**N = 30**	**N = 60**
Age (years)	Mean (SD)	39.9 (9.37)	39.8 (8.75)	39.9 (8.99)	41.2 (9.86)	40.5 (9.27)	40.8 (9.50)
	Range	19-53	23-53	19-53	20-53	24-53	20-53
Height (cm)	Mean (SD)	165.0 (6.45)	165.8 (4.78)	165.4 (5.64)	164.8 (6.17)	164.5 (6.16)	164.6 (6.11)
	Range	152-184	153-178	152-184	151-177	154-176	151-177
Weight (kg)	Mean (SD)	65.78 (10.64)	63.34 (8.97)	64.56 (9.83)	63.24 (10.44)	61.67 (9.68)	62.46 (10.02)
	Range	50.4-94.8	50.2-81.4	50.2-94.8	50.2-84.4	50.4-83.1	50.2-84.4
BMI (kg/m^2^)	Mean (SD)	24.12 (3.14)	23.06 (3.19)	23.59 (3.18)	23.25 (3.26)	22.73 (2.85)	22.99 (3.05)
	Range	18.74-29.41	18.22-28.95	18.22-29.41	18.68-29.73	18.68-29.73	18.68-29.73
Sex	Male	30 (100%)	30 (100%)	60 (100%)	30 (100%)	30 (100%)	60 (100%)
Race	Asian	30 (100%)	30 (100%)	60 (100%)	30 (100%)	30 (100%)	60 (100%)
Ethnicity	Indian (Indian subcontinent)	30 (100%)	30 (100%)	60 (100%)	30 (100%)	30 (100%)	60 (100%)

Sequence 1a: single dose of 80/480 mg AL tablet (80 mg artemether/480 mg lumefantrine) // single dose of four tablets of AL (each 20 mg artemether/120 mg lumefantrine).

Sequence 2a: single dose of four tablets of AL (each 20 mg artemether/120 mg lumefantrine) // single dose of 80/480 mg AL tablet (80 mg artemether/480 mg lumefantrine).

Sequence 1b: single dose of 60/360 mg AL tablet (60 mg artemether/360 mg lumefantrine) // single dose of three tablets of AL (each 20 mg artemether/120 mg lumefantrine).

Sequence 2b: single dose of three tablets of AL (each 20 mg artemether/120 mg lumefantrine) // single dose of 60/360 mg AL tablet (60 mg artemether/360 mg lumefantrine).

### Pharmacokinetics and bioequivalence

**Figure 1:**
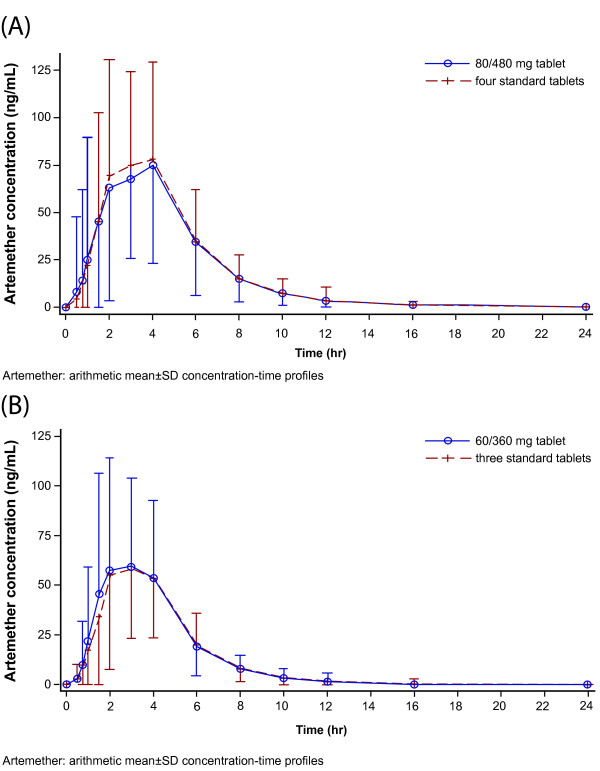
**Artemether: arithmetic mean ± SD concentration-time profiles.** Mean plasma concentration-time profiles for comparison of **(A)** 80/480 mg tablet and four standard tablets **(B)** 60/360 mg tablet and three standard tablets.

**Figure 2:**
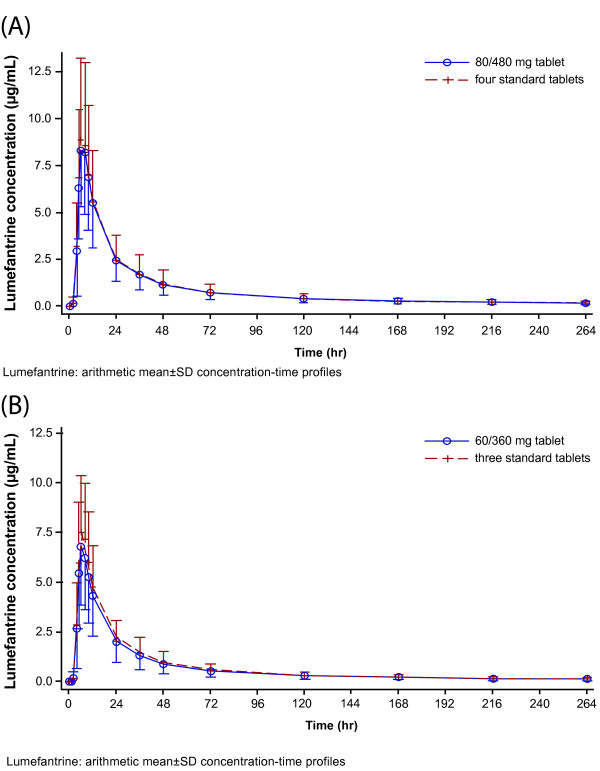
**Lumefantrine: arithmetic mean ± SD concentration-time profiles.** Mean plasma concentration-time profiles for comparison of **(A)** 80/480 mg tablet and four standard tablets **(B)** 60/360 mg tablet and three standard tablets.

**Figure 3:**
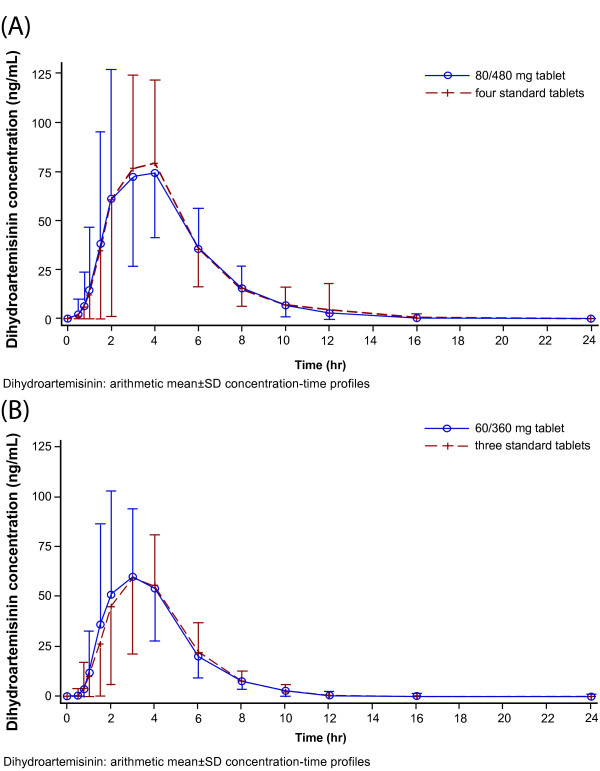
**Dihydroartemisinin: arithmetic mean ± SD concentration-time profiles.** Mean plasma concentration-time profiles for comparison of **(A)** 80/480 mg tablet and four standard tablets **(B)** 60/360 mg tablet and three standard tablets.

The mean plasma concentration-time profiles of artemether, lumefantrine, and DHA of the novel 80/480 mg and 60/360 mg tablet formulations were superimposable with their corresponding standard tablet formulations (Figure 1, Figure 2 and Figure 3).

Following the single dose administration, artemether was absorbed rapidly with median T_max_ of three hours for both 80/480 mg tablet and four standard tablets (Table 2). A mean C_max_ of 113 ng/mL was obtained for both the 80/480 mg and four standard tablets. Mean AUC_last_ was 389 ng h/mL *versus* 408 ng∙h/mL and mean AUC_inf_ was 408 ng∙h/mL *versus* 443 ng∙h/mL for the 80/480 mg tablet compared to four standard tablets, respectively. The mean T_1/2_ was in the range of 2.30-2.51 h. Lumefantrine was absorbed with median T_max_ of 6.00 h. A mean C_max_ of 8.92 μg/mL and 9.49 μg/mL was observed for the 80/480 mg tablet and four standard tablets, respectively. Mean AUC_last_ for the 80/480 mg tablet was 236 μg∙h/mL compared to 243 μg∙h/mL for the dose of four standard tablets. Mean AUC_inf_ for the 80/480 mg tablet was 261 μg∙h/mL compared to 277 μg∙h/mL for the dose of four standard tablets. The mean T_1/2_ was in the range of 117–120 h for the 80/480 mg and four standard tablets. For DHA, a median T_max_ of 3.00 h was observed for the 80/480 mg and four standard tablets. Mean C_max_ was 107 ng/mL and 110 ng/mL for the 80/480 mg tablet and four standard tablets, respectively. Mean AUC_last_ for the 80/480 mg tablet and the dose of four standard tablets were 376 ng∙h/mL and 386 ng∙h/mL, respectively. Corresponding AUC_inf_ values were 397 ng∙h/mL and 397 ng∙h/mL, respectively. The mean T_1/2_ for DHA was in the range of 1.87-1.98 h.

**Table 2:** Summary statistics of PK parameters

**Analyte**	**PK parameter**^#^	**80/480 mg tablet**	**Four standard tablets**	**60/360 mg tablet**	**Three standard tablets**
Artemether	C_max_ (ng/mL)	113 ± 69.5	113 ± 58.9	91.2 ± 54.4	82.4 ± 42.0
		[n = 58]	[n = 59]	[n = 57]	[n = 60]
	AUC_last_ (ng·h/mL)	389 ± 207	408 ± 198	280 ± 171	267 ± 127
		[n = 58]	[n = 59]	[n = 57]	[n = 60]
	AUC_inf_ (ng·h/mL)	408 ± 209	443 ± 202	315 ± 173	301 ± 123
		[n = 55]	[n = 53]	[n = 50]	[n = 49]
	T_max_ (h)	3.00 (1.00;8.00)	3.00 (0.75;12.0)	3.00 (1.00;6.02)	3.00 (0.75;6.00)
		[n = 58]	[n = 59]	[n = 57]	[n = 60]
	T_1/2_ (h)	2.30 ± 1.11	2.51 ± 2.01	1.89 ± 0.724	1.83 ± 0.834
		[n = 55]	[n = 53]	[n = 50]	[n = 49]
Lumefantrine	C_max_ (μg/mL)	8.92 ± 3.18	9.49 ± 4.41	7.26 ± 2.84	8.16 ± 2.86
		[n = 58]	[n = 59]	[n = 57]	[n = 59]
	AUC_last_ (μg·h/mL)	236 ± 93.0	243 ± 122	181 ± 83.9	200 ± 85.1
		[n = 58]	[n = 58]	[n = 56]	[n = 56]
	AUC_inf_ (μg·h/mL)	261 ± 106	277 ± 146	200 ± 96.7	221 ± 96.2
		[n = 56]	[n = 51]	[n = 53]	[n = 54]
	T_max_ (h)	6.00 (4.00;10.0)	6.00 (5.00;12.0)	6.00 (5.00;12.0)	6.00 (5.00;10.0)
		[n = 58]	[n = 59]	[n = 57]	[n = 59]
	T_1/2_ (h)	117 ± 37.9	120 ± 43.9	104 ± 46.5	111 ± 61.6
		[n = 58]	[n = 56]	[n = 56]	[n = 56]
Dihydroartemisinin	C_max_ (ng/mL)	107 ± 53.7	110 ± 50.7	83.6 ± 41.0	78.5 ± 36.8
		[n = 58]	[n = 59]	[n = 57]	[n = 59]
	AUC_last_ (ng·h/mL)	376 ± 126	386 ± 130	262 ± 98.7	256 ± 93.2
		[n = 58]	[n = 59]	[n = 57]	[n = 59]
	AUC_inf_ (ng·h/mL)	397 ± 122	397 ± 130	290 ± 95.2	284 ± 93.6
		[n = 56]	[n = 57]	[n = 47]	[n = 48]
	T_max_ (h)	3.00 (1.00;8.00)	3.00 (1.50;12.0)	3.00 (1.50;10.0)	3.00 (1.00;6.00)
		[n = 58]	[n = 59]	[n = 57]	[n = 59]
	T_1/2_ (h)	1.98 ± 1.00	1.87 ± 0.789	1.57 ± 0.420	1.52 ± 0.365
		[n = 56]	[n = 57]	[n = 47]	[n = 48]

^#^All PK parameter values are presented as mean ± SD except T_max_ which is presented as median (range); number of subjects is given in square brackets.

For 60/360 mg and three standard tablet formulations, artemether was absorbed rapidly with median T_max_ of 3.00 h (Table 2). C_max_ for 60/360 mg tablet and a dose of three standard tablets were 91.2 ng/mL and 82.4 ng/mL, respectively. For the 60/360 mg tablet compared to the dose of three standard tablets, AUC_last_ and AUC_inf_ were 280 ng∙h/mL *versus* 267 ng∙h/mL, and 315 ng∙h/mL *versus* 301 ng∙h/mL, respectively. The mean T_1/2_ of artemether ranged between 1.83 and 1.89 h. Lumefantrine was absorbed with median T_max_ of 6 h. Mean C_max_ for 60/360 mg tablet and the dose of three standard tablets was 7.26 μg/mL and 8.16 μg/mL, respectively. Mean AUC_last_ for the 60/360 mg tablet compared to the dose of three standard tablets were 181 μg∙h/mL and 200 μg∙h/mL, respectively. Corresponding mean AUC_inf_ values were 200 μg∙h/mL and 221 μg∙h/mL, respectively. The mean T_1/2_ was in the range of 104–111 h for both treatments. For DHA, the median T_max_ was 3.00 h for the 60/360 mg and three standard tablets. C_max_ for 60/360 mg tablet and the dose of three standard tablets was 83.6 ng/mL and 78.5 ng/mL, respectively. Mean AUC_last_ for the 60/360 mg tablet and the dose of three standard tablets were 262 ng∙h/mL and 256 ng∙h/mL, respectively. Corresponding AUC_inf_ values were 290 ng∙h/mL and 284 ng∙h/mL, respectively. The mean T_1/2_ for DHA was 1.52 to 1.57 h for the 60/360 mg and three standard tablets.

Adjusted geometric means and associated 90% CI of C_max_ and AUC_last_ for artemether and lumefantrine (Table 3) were contained within the interval (0.80, 1.25). Additionally, adjusted geometric means and associated 90% CI of secondary PK parameters, including AUC_inf_ for artemether and lumefantrine, as well as C_max_, AUC_last_, and AUC_inf_ for DHA, were also contained within the interval (0.80, 1.25).

**Table 3:** Artemether, lumefantrine and dihydroartemisinin: 90% CI for relevant PK parameters

**Formulation**	**Analyte**	**PK**	**Geometric mean ratio**		
		**variable**	**Estimate**	**Lower**	**Upper**
				90% CI*	90% CI*
**80/480 mg vs four standard tablets**	Artemether	AUC_last_	0.95	0.89	1.01
		C_max_	0.97	0.89	1.06
		AUC_inf_	0.95	0.89	1.02
	Lumefantrine	AUC_last_	1.00	0.93	1.08
		C_max_	0.97	0.89	1.05
		AUC_inf_	1.00	0.92	1.09
	Dihydroartemisinin	AUC_last_	0.98	0.93	1.04
		C_max_	0.96	0.88	1.05
		AUC_inf_	1.00	0.95	1.05
**60/360 mg *vs *three standard tablets**	Artemether	AUC_last_	1.02	0.94	1.10
		C_max_	1.06	0.95	1.18
		AUC_inf_	1.00	0.93	1.09
	Lumefantrine	AUC_last_	0.89	0.83	0.96
		C_max_	0.87	0.81	0.94
		AUC_inf_	0.89	0.83	0.96
	Dihydroartemisinin	AUC_last_	1.00	0.94	1.07
		C_max_	1.02	0.94	1.12
		AUC_inf_	1.03	0.97	1.10

*CI confidence interval.

Model: The log transformed PK parameter data were analyzed using a linear model with treatment, sequence, period and subject nested within sequence as fixed factors.

Nine subjects (80/480 mg tablet or four standard tablets: four subjects; 60/360 mg tablet or three standard tablets: five subjects) experienced a total of ten AEs during the study period (Table 4). Seven AEs were not suspected to be related to the study medication and three AEs (two cases of vomiting and one case of pyrexia) were suspected to be related to the study medication. The affected system organ class (SOC) were injury, poisoning and external events (navicular bone fracture, radius fracture, excoriation), investigations (blood lactate dehydrogenase increased, blood creatine phosphokinase increased), gastrointestinal disorders (vomiting) and general disorders and administration site conditions (pyrexia). Most AEs were mild to moderate in severity. No death and no SAE were reported during the study. No clinically significant trends in any clinical laboratory measurement and no changes in vital signs were observed during the study. One subject who received four tablets had corrected QT interval using Friderica’s formula (QTcF) and corrected QT interval using Bazett’s formula (QTcB) increases of 44 and 62 msec, respectively, from baseline (day -1) 2 h post-dose on day 1. This appeared to be a reflection of the subject’s high pre-drug exposure variability in QTcF with values of 403, 358, and 422 msec at screening, baseline, and pre-dose day 1, respectively, and with a 402 msec 2 h post-dose day 1 QTcF value. In the period 2 dosing (novel 80/480 mg tablet) there was minimal change from baseline in either QTcF or QTcB with similar drug exposure to AL. The investigator did not report this corrected QT (QTc) change as an adverse event. Based on the overall review, the QTcF and QTcB changes were considered not drug related or clinically significant but an artifact of QTc variability in the subject.

**Table 4:** Subjects with adverse events by body system and preferred term

		**80/480 mg tablet**	**Four standard tablets**	Total*	**60/360 mg tablet**	**Three standard tablets**	Total**
**Body system**	**Preferred term**	**N = 58**	**N = 60**	**N = 60**	**N = 57**	**N = 60**	**N = 60**
		**n (%)**	**n (%)**	**n (%)**	**n (%)**	**n (%)**	**n (%)**
Any body system	Total	1 (1.7)	3 (5.0)	4 (6.7)	1 (1.8)	4 (6.7)	5 (8.3)
Gastrointestinal disorders	Vomiting	0 (0.0)	1 (1.7)	1 (1.7)	0 (0.0)	1 (1.7)	1 (1.7)
General disorders and administrative site conditions	Pyrexia	0 (0.0)	0 (0.0)	0 (0.0)	0 (0.0)	1 (1.7)	1 (1.7)
Injury, poisoning and procedural complications	Excoriation	0 (0.0)	0 (0.0)	0 (0.0)	1 (1.8)	0 (0.0)	1 (1.7)
	Radius fracture	0 (0.0)	0 (0.0)	0 (0.0)	0 (0.0)	1 (1.7)	1 (1.7)
	Navicular bone fracture	0 (0.0)	1 (1.7)	1 (1.7)	0 (0.0)	0 (0.0)	0 (0.0)
Investigations	Blood creatine phosphokinase increased	0 (0.0)	1 (1.7)	1 (1.7)	0 (0.0)	1 (1.7)	1 (1.7)
	Blood lactate dehydrogenase increased	1 (1.7)	1 (1.7)	2 (3.3)	0 (0.0)	0 (0.0)	0 (0.0)

*An overall summary for all subjects treated with 80/480 mg tablet or four standard tablets.

**An overall summary for all subjects treated with 60/360 mg tablet or three standard tablets.

A subject with multiple adverse events within a system organ class is counted only once in the total row.

## Discussion

The efficacy of AL may be influenced by dosage accuracy, adherence to the treatment regimen, and food intake accompanying the treatment. The reduction in pill burden may promote adherence to treatment compared with existing formulations, and may have a significant impact on the effective treatment of malaria and subsequently a decrease in parasite transmission. The current study compared the systemic exposure to artemether, DHA and lumefantrine from novel AL single tablets of 80/480 mg and 60/360 mg to their respective number of standard market tablets.

Both novel tablets of AL met the prespecified criteria for bioequivalence as the geometric mean ratios were approximately one and the 90% CIs for the geometric mean ratios for the primary pharmacokinetic endpoints of AUC_last_ and C_max_ for artemether and lumefantrine were contained within the acceptance interval (0.80, 1.25) for bioequivalence. This demonstrates that rate and extent of absorption of both components of AL from the novel formulations are comparable to that from the standard tablets. In addition, other PK parameters, including those for DHA, also met the criterion of bioequivalence for C_max_, AUC_last_ and AUC_inf_ in both treatment comparisons and thus provide further evidence for the bioequivalence of the novel tablet formulations of AL and the respective number of standard tablets.

This bioequivalence study was designed as a single dose, open-label, randomized study with a two-period, within formulation crossover design in line with both FDA and EU guidelines [9]. Potential sources of variation (within-subject, between-subject, and subject-by-formulation interaction) and the pharmacokinetic properties of the active substances were considered. Additionally, the sample size was adequate to show bioequivalence. The four formulations were administered with food as recommended in the approved labelling for Coartem® as a meal is known to enhance the bioavailability [10] of artemether and lumefantrine compared with the fasted state [11].

The PK evaluations for all the formulations did not reveal any noteworthy deviation in the PK parameters in this study from those in prior studies in healthy subjects [8,12]. C_max_, AUC_last_, and AUC_inf_ were similar between the two treatments (in both comparisons) for artemether and lumefantrine. The DHA exposure in the treatment groups was also similar. The percentage extrapolated area for calculation of AUC_inf_ was < 20% indicating that the study sampling schemes for both artemether and lumefantrine were adequate. The disposition of artemether, DHA and lumefantrine was in line with previously published data [12-14], confirming that the concentration-time characterization and washout period of five weeks were adequate, and avoided potential crossover effects.

Overall, the novel and standard AL tablet formulations were well tolerated. The overall incidence of AEs was similar across the treatment groups and in both the sequences. There was no SAE, death or discontinuation due to the study drug.

Another point worth emphasizing is that the food effect was similar across all formulations. In particular, the corresponding T_max_ for both artemether and lumefantrine remained the same, irrespective of the number and nature of tablets (novel *versus* standard tablets) and strength (20/120 mg *versus* 80/480 mg *versus* 60/360 mg), which indicate that the *in vivo* dissolution of artemether and lumefantrine from the novel tablets was not impacted by these modifications. The results of this study support the use of the novel tablets developed as they are easy to administer and may improve adherence in the treatment of uncomplicated *P*. *falciparum* malaria.

## Competing interests

GL, PB, JPJ, SK, YC and DSS are full-time employees of Novartis. HD has no competing interest.

## Authors’ contributions

GL and DS designed the research study, oversaw the study conduct, analysed the data, and wrote the first draft of the manuscript. PB, JPJ, SK, and YC designed the research study and analysed the data. HD collected and analysed data, and was involved in subject care. All authors participated in the preparation of the manuscript. All authors read and approved the final manuscript.
